# Long-term Effect of Split Iron Dextran/Hemoglobin Supplementation on Erythrocyte and Iron Status, Growth Performance, Carcass Parameters, and Meat Quality of Polish Large White and 990 Line Pigs

**DOI:** 10.1007/s12011-019-01950-w

**Published:** 2019-11-08

**Authors:** Mateusz Szudzik, Paweł Lipiński, Aneta Jończy, Rafał Mazgaj, Marek Pieszka, Marian Kamyczek, Ewa Smuda, Rafał R. Starzyński

**Affiliations:** 1 Department of Molecular Biology, Institute of Genetics and Animal Breeding PAS, ul. Postępu 36a, 05-552 Magdalenka, Jastrzębiec Poland; 2 Department of Animal Nutrition & Feed Science, National Research Institute of Animal Production, Kraków, Poland; 3 Pig Hybridization Centre, National Research Institute of Animal Production, Pawłowice, Poland

**Keywords:** Heme, Iron deficiency anemia, Pig, Supplementation, Slaughter performance, Meat quality

## Abstract

Heme is an efficient dietary iron supplement applied in humans and animals to prevent iron deficiency anemia (IDA). We have recently reported that the use of bovine hemoglobin as a dietary source of heme iron efficiently counteracts the development of IDA in young piglets, which is the common problem in pig industry. Here, we used maternal Polish Large White and terminal sire breed (L990) pigs differing in traits for meat production to evaluate the long-term effect of split supplementation with intramuscularly administered small amount of iron dextran and orally given hemoglobin on hematological indices, iron status, growth performance, slaughter traits, and meat quality at the end of fattening. Results of our study show that in pigs of both breeds split supplementation was effective in maintaining physiological values of RBC and blood plasma iron parameters as well as growth performance, carcass parameters, and meat quality traits. Our results prove the effectiveness of split iron supplementation of piglets in a far-reach perspective.

## Introduction

Early postnatal iron deficiency anemia (IDA) is a widespread pathology in livestock affecting especially suckling piglets [1, 2]. The common cause of iron deficiency in newborn piglets is a striking imbalance between high iron demand and inadequate iron supply. Huge iron requirements during the first few weeks after birth (7–16 mg Fe/piglet/day) [2, 3] result from the selection of piglets for a large litter size, high birth weight, rapid growth and in consequence greater blood volume, and increased red blood cells (RBC) count. Considering that RBC are the largest reservoir of iron in the body [2], meeting iron requirements for erythropoiesis from natural iron-deficient sources such as hepatic iron stores [2, 4] and sow’s milk [5, 6] is unreachable in suckling piglets [7]. Without iron supplementation piglets regularly become anemic within 2 weeks postpartum [2].

Intramuscular administration of large amount of iron dextran (FeDex) on days 3 to 6 postpartum is current practice in the swine industry, rectifying the hematological status of piglets [8–11]. However, routinely applied dose of 200 mg Fe per piglet in the form of a single intramuscular injection of FeDex seems not being efficiently metabolized and detoxified [8]. Moreover, we have previously reported that high iron parenteral supplementation with FeDex induces expression of hepcidin (Hepc) [8, 12, 13], a 25-amino acid peptide hormone, the master regulator of the systemic iron homeostasis in vertebrates [14]. It is well established that high Hepc may perturb body iron homeostasis by inhibiting both duodenal iron absorption and iron recycling by macrophages of the reticuloendothelial system (RES) that catabolize heme contained in phagocytosed senescent RBC [15]. In consequence of this two-way hepcidin-dependent regulation, availability of iron for erythropoiesis is strongly decreased.

To minimize both toxicity of supplemental iron and its capacity to induce hepcidin synthesis, we proposed several modifications of routine procedure of FeDex administration to piglets [8, 12]. Furthermore, considering that heme preparations have been successfully used to prevent and treat IDA in humans [16–18], dogs [19], and pigs [20, 21], we used bovine hemoglobin as heme iron supplement. We showed that oral supplementation of piglets with hemoglobin rescues them from severe IDA observed in non-supplemented animals [22]. However, to strengthen the curative effect of dietary hemoglobin, we successfully used a split iron supplementation regime involving additional supportive injection of a small amount of FeDex on day 3 after birth. This modified procedure has been shown to better cover iron needs of 28-day-old piglets, i.e., at the age of weaning [22]. Indeed, most studies dealing with iron supplementation procedures in piglets evaluate their efficacy in animals during the period from birth to weaning [1, 2]. However, a final, far-reaching goal of iron supplementary procedures in pig breeding is to assure a permanent and durable iron balance in pigs until slaughter. Therefore, in this study, we evaluated a long-term effect of split (FeDex/hemoglobin) iron supplementation on RBC status, iron content in tissues (including skeletal muscles), and its impact on growth performance, carcass parameters, and meat quality traits of ~ 180-day-old (slaughter age) Polish Large White (PLW) and 990 line (L990) pigs. We used in our experiment these two Polish breeds considering their different characteristics of meat performance and distinct use in crossbreeding program in Poland [23]. In contrast to PLW, a typical maternal breed, L990 is exploited as a terminal sire breed [24]. Importantly, we compared our experimental supplementation with the procedure considered a gold standard in the therapy of IDA in pigs, involving intramuscular injection of high amount of iron in the form of FeDex.

We provide evidence that the experimental split iron supplementation of piglets, which is not fully effective at weaning, does not entail negative consequences for the maintenance of RBC indices, systemic iron status, growth performance, and meat quality of pigs at the end of fattening.

## Materials and Methods

### Animals and Experimental Design

Experiment was conducted at the Pig Hybridization Centre in Pawłowice (National Research Institute of Animal Production, Balice, Poland). The experimental procedures used in this study were in compliance with the EU guidelines for the care and handling of research animals (EU Directive 2010/63/EU for animal experiments). According to Polish legislation, tests on animals using feed additives and supplements (including iron supplements such as FeSO_4_, hemoglobin, dried erythrocytes, plasma, blood) are not considered research procedures and therefore do not interfere with animal welfare. Therefore, ethical permission is not required. A total of 28 Polish Large White (PLW) and 990 line (L990) piglets (males) born from 6 sires (3 PLW and 3 L990) and 8 dams (4 PLW and 4 L990) from different litters, housed in standard conditions (70% humidity and a temperature of 22 °C in cages with straw bedding), were used. Until weaning (day 28 after birth) sows were allowed to nurse their piglets and piglets had no access to the sows’ feed. The Prestarter Wigor 1 Plus feed (containing 238 mg Fe/kg as estimated by flame spectrometry) was offered to piglets from day 5 to day 45 after birth. Piglets from both breeds were allotted into the following 4 experimental groups (7 piglets per group) on the basis of balanced body weight at day 3 after birth: PLW and L990, piglets intramuscularly injected with 100 mg Fe/kg b.w. in the form of iron dextran, FeDex (Ferran 100, Vet-Agro, Lublin, Poland) on day 3 after birth (supplementation routinely applied to piglets at the Pig Hybridization Centre in Pawłowice); PLW and L990, piglets intramuscularly injected with 40 mg Fe/kg b.w in the form of FeDex on day 3, and supplemented orally from day 3 to day 45 with bovine hemoglobin (Bovogen, East Keilor, Australia) added to the feed in the proportion 38 g hemoglobin per 1 kg of feed (split supplementation). The final total iron content in this mixture, assessed by flame atomic absorption spectroscopy was 612 mg Fe/kg. The mean daily consumption of feed per piglet and the respective calculated iron intake were monitored in the 4 experimental groups and did not differ significantly between animals (data not shown). After 45th day of life, piglets were fed with the following typical feeds offered to pigs during intensive fattening: starter (up to body weight 30 kg containing 150 mg Fe/kg feed), grower (until body weight 70 kg containing 100 mg Fe/kg feed), and finisher (until the day of slaughter containing 80 mg Fe/kg feed).

After reaching the slaughter weight—110 kg (on day ~ 180 after birth)—animals were transported to the slaughterhouse belonging to National Research Institute of Animal Production, Pig Hybridization Centre, and slaughtered by exsanguination after being electric stunned, then scalded, dehaired, eviscerated, and split down the midline according to commercial procedures.

### Biological Sample Collection

Blood was drawn on day 28 after birth by venipuncture of the jugular vein (*vena jugularis externa*) and at slaughter using syringe with Li-Heparin (S-Monovette® with S-Monovette-Needle®). The blood samples were centrifuged (1200×*g*, 10 min, 4 °C) to separate the plasma. Plasma samples were immediately aliquoted and stored at − 80 °C. Samples of the liver, spleen, kidney, heart, and skeletal muscles—*musculus longissimus thoracis et lumborum*, *musculus gluteus maximus*—were collected, rinsed with PBS, and then stored at − 80 °C until they were used for biochemical analyses.

### Measurement of Red Blood Cell Indices and Iron Parameters in the Blood Plasma

RBC indices were measured using ADVIA 2010 analyzer (Siemens, Germany). Iron concentration in the blood plasma and total iron binding capacity (TIBC) were determined by colorimetric measurement of the absorbance of the iron-chromasurol complex at 630 nm according to the manufacturer’s protocol (Biomaxima S.A., Poland). Percent of transferrin saturation (TSAT) was then calculated according to the following formula: TSAT = [plasma iron/TIBC] × 100.

### Quantitative Measurement of Non-heme Iron Content in Tissues

The non-heme iron content of the liver, spleen, kidney, heart, and skeletal muscles (*gluteus maximus and longissimus thoracis et lumborum*) were determined using colorimetric assay as described previously [25].

### Quantitative Measurement of Heme Iron Content in Longissimus Thoracis et Lumborum Muscle

The heme content of formic acid–solubilized tissues was determined spectrophotometrically at 398 nm using hemin standards prepared in formic acid and a molar absorption coefficient of 1.5 × 10^5^ M^−1^ cm^−1^ [26].

### Real-time Quantitative RT-PCR Analysis of Hepatic Hepcidin mRNA Abundance and Blood Plasma Hepcidin-25 Quantification

Hepatic hepcidin mRNA levels were measured by a real-time quantitative RT-PCR of cDNA derived from specific transcript in a Light Cycler U96 (Roche Diagnostics, Mannheim, Germany), using the pair of following primers: forward (5′-3′) AAGACAGCTCACAGACCTCC, reverse (5′-3′) CTACGTCTTGCAGCACATCC. The amplified products were detected using SYBR Green I (Roche Diagnostics) as described previously [22]. For data analysis Light Cycler U96 Software was used. Expression was quantified relative to that of a control transcript encoding the *glutathione reductase* (GSR) Forward (5′-3′) CACAGCTCCTCACATCCTGA, Reverse (5′-3′) GGGCAATTCTTCCAGCTGAA.

Measurement of hepcidin-25 level in the blood plasma was performed as described previously for porcine plasma samples [13] by a combination of weak cation exchange chromatography and time-of-flight mass spectrometry (WCX-TOF MS) [27].

### Analysis of the Meat Quality and Production Traits

Immediately after slaughter, blood samples were collected as described for 28-day-old piglets. Samples of the duodenum, liver, spleen, kidney, and muscles were collected for further analyses. All carcass parameters were tested according to the methodology applied in Polish Pig Testing Stations as described by Różycki (1996) [28]. The following parameters were registered: average slaughter age, average daily gain (standardized on day 180 of age), slaughter yield, body weight at slaughter, half carcass weight, dressing percentage, meatiness percentage using CGM Sydel apparatus. Meat quality parameters such as loin “eye” area (LEA), backfat thickness (FT), and color/brightness were measured. On hot, hanging right carcass sides using slide caliper backfat thickness at points FTI to FTV were measured Borzuta (1998) [29]. For the LEA measurement, the cut has been made between the last thoracic vertebra and the first lumbar vertebrae and the contour of the muscle was done for planimeter assessment. Color/brightness of *longissimus thoracis et lumborum* muscle using MINOLTA CHROMA METERS CR 400. The instrument was calibrated against a standard white plate (8-mm-diameter aperture, d/0 illumination system, D65 illuminant and a 2° standard observer angle) determining L*color component (brightness) parameter was measured. Each measurement was performed 6 times. The mean value was used as the result.

### Statistical Analysis

Results were statistically analyzed with one-way analysis of variance (ANOVA), and Tukey-Kramer post hoc test using GraphPad Prism software (GraphPad, San Diego, CA, USA). The treatment was the fixed effect, and sires were the random effect in the statistical model. *P* ≤ 0.05 was considered significant. Data are presented as mean values ± SE. Experiments were designed in two replicates.

## Results and Discussion

Most iron supplementation procedures in piglets are designed to provide exogenous iron as early as on day 3–6 after birth and thus to prevent the development of IDA during first few weeks of life. Indeed, this period is critical for the maintenance of iron balance because of extremely high iron requirements to sustain hemoglobin synthesis in erythroid cells. Without supplementation, piglets may also show an impaired functioning of the immune system resulting in a greater susceptibility to infectious and parasitic diseases [30]. Most studies dealing with iron supplementation strategies in suckling piglets consider their effectiveness by evaluating RBC indices and iron status of piglets at weaning (in 4-week-old animals) [8–11] To our knowledge, little attention has been paid to the effect of pre-weaning iron supplementation on the status of this microelement in pigs during the post-weaning period [31]. Meanwhile, it seems that in a long-term perspective iron repletion in pigs is important not only for their health but also for their growth performance and nutritional value of pork.

**Fig. 1:**
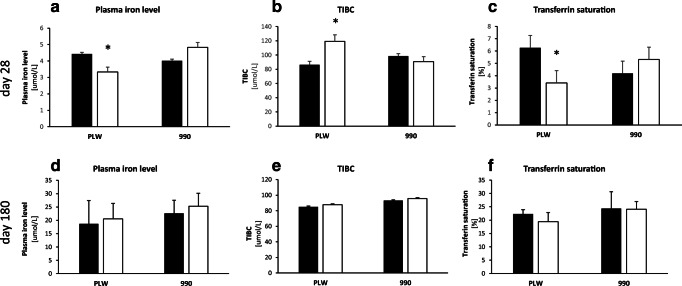
Iron status in the blood plasma of PLW and L990 in 28-day-old and ~ 180-day-old pigs receiving various iron supplementations. 28-day-old: (**a**) blood plasma iron; (**b**) total iron binding capacity (TIBC); (**c**) transferrin saturation; ~ 180-day-old: (**d**) blood plasma iron; (**e**) total iron binding capacity (TIBC); (**f**) transferrin saturation. Values are expressed as the means ± SE for plasma samples obtained from 7 pigs from each experimental group. Solid bars, piglets supplemented with high-dose FeDex; open bars, piglets receiving split FeDex/hemoglobin supplementation

**Fig. 2:**
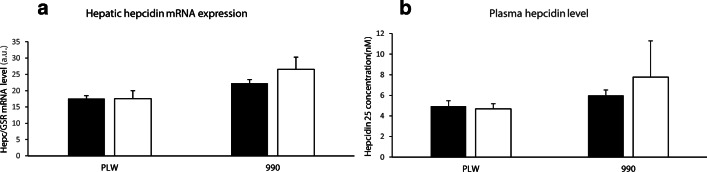
Hepatic hepcidin mRNA expression and plasma hepcidin concentrations of PLW and L990 ~ 180-day-old pigs receiving various iron supplementations: (**a**) hepatic hepcidin mRNA expression; (**b**) plasma hepcidin concentrations. Solid bars, piglets supplemented with high-dose FeDex; open bars, piglets receiving split FeDex/hemoglobin supplementation

**Fig. 3:**
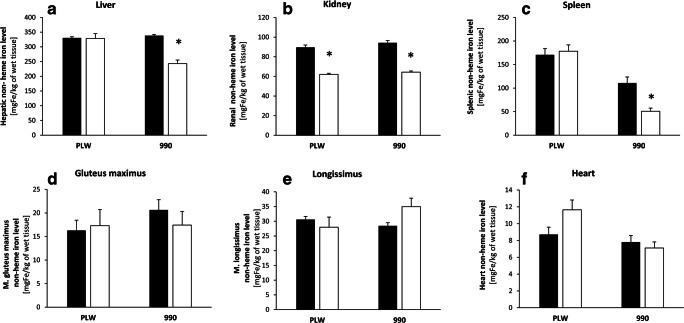
Comparison of the tissular non-heme iron content in PLW and L990 ~ 180-day-old pigs receiving various iron supplementations. Tissue non-heme iron content was measured in following tissues: (**a**) liver; (**b**) kidney; (**c**) spleen; (**d**) *gluteus maximus* muscle; (**e**) *longissimus thoracis et lumborum* muscle; and (**f**) heart. Values are expressed as the means ± SE for tissue samples obtained from pigs of each group (*N* = 7). *P < 0.05, significant differences between pigs receiving split and routine supplementation within a given breed. Solid bars, piglets supplemented with high-dose FeDex; open bars, piglets receiving split FeDex/hemoglobin supplementation

In the present study, we first aimed at evaluating the impact of an early split iron supplementation in piglets on both RBC and iron plasma parameters at the end of fattening, i.e., in approximately 180-day-old PLW and L990 pigs (Table 1 and Fig. 1). We hypothesized that although piglets receiving a combined supplementation are not fully replete with iron at weaning [22, 32 and this study—Table 1], during the post-weaning period they can accommodate iron absorption to sustain growth performance and iron content in tissues. Accordingly, it has been demonstrated that a few months pigs highly express apical (divalent metal transporter 1) and basolateral (ferroportin) iron transporters not only on duodenal enterocytes but also on colonocytes [33]. Concentration of hemoglobin in 28-day-old piglets receiving split supplementation indicates that depending on various cut-off values, these animals are slightly below [34] or above [35] the border line of anemia (Table 1). It is noteworthy that blood plasma iron parameters indicate slight iron deficiency but only in PLW piglets (Fig. 1a-c). In contrast, piglets supplemented with high amount of FeDex definitely exhibit a non-anemic phenotype (Table 1). In our previous study we demonstrated that 28-day-old piglets supplemented exclusively with a single, low dose of FeDex (i.e., a parenteral component of split supplementation used in this study) are definitely iron deficient and show clear symptoms of IDA [13]. Assuming that IDA in these animals at weaning is far advanced, we considered them not suitable for investigating long-term effect of preweaning iron supplementation. Interestingly, at weaning piglets from two analyzed breeds within a given supplementation group show very similar RBC status, except higher level of HGB in PLW animals (Table 1). In contrast, at the end of fattening, L990 pigs display significantly higher values of the majority of RBC indices such as RBC count, hemoglobin concentration, and hematocrit values compared with PLW animals (Table 1). Most importantly, RBC parameters in finishing animals of both breeds receiving either routine or split supplementation do not differ significantly (Table 1). Similarly, iron status in the blood plasma of PLW and L990 adult pigs is comparable regardless supplementation procedure (Fig. 1d-f). Consequently, hepcidin mRNA expression as well as blood plasma concentration of hepcidin-25 in adult pigs shows no statistically significant differences neither between pig breeds nor between supplementation groups (Fig. 2). These results clearly indicate that piglets receiving split supplementation and showing slightly anemic RBC status at weaning, during post-weaning period are able to improve RBC parameters, stabilize them within the physiological range [32, 36–38], and correct their iron status. This recovery attests a very efficient process of iron absorption from high iron–containing diets given to growing and finishing pigs (see the “Materials and Methods” section). Appropriate values of RBC indices are not always associated with iron repletion in tissues because erythroid compartment is on the first line of iron demand consuming major part of iron supply in the body [39]. Therefore, we measured iron content in tissues such as the liver, spleen, kidney, skeletal muscles, and heart and found statistically significant differences between animals from two supplementation groups in the kidney (lower iron content in pigs receiving split supplementation in both PLW and L990 breeds), in the spleen (lower iron content in L990 pigs supplemented with FeDex and hemoglobin), and in the liver (lower iron content in L990 pigs supplemented with hemoglobin) (Fig. 3). Interestingly, splenic iron content was significantly lower in L990 than in PLW pigs regardless the supplementation and we hypothesized that this is connected with overall higher iron demand for erythropoiesis in L990 pigs showing higher RBC status compared with PLW animals (Table 1). Hemoglobin contained in erythrocytes accounts for the largest pool of heme and, consequently, iron. Bone marrow erythroblasts acquire more than 80% of plasma iron [39]. Therefore, higher RBC status of L990 piglets is associated with higher iron demand to maintain adequate erythropoiesis in these animals. According to Michalska et al. 2006 [40], pigs of L990 breed (a terminal sire breed) are also characterized by faster growth rate than animals belonging to PLW breed (maternal breed) and it could be an additional factor of greater iron requirements of these animals.

**Table 1:** Hematological indices of PLW and L990 28- and ~ 180-day-old pigs receiving various iron supplementations

	Parameter	RBC (× 10^6^/μL)		HGB (g/dL)		HCT (%)		MCV (fL)	
Breed ↓	Age (days) → Supplementation ↓	28	180	28	180	28	180	28	180
PLW	Iron dextran	6.5 ± 0.11	7.49 ± 0.18	12.02 ± 0.50	12.34 ± 0.38	26.12 ± 1.37	39.09 ± 1.24	43.55 ± 2.22	52.14 ± 0.85
	Split	6.05 ± 0.49	7.24 ± 0.41	8.04 ± 0.58**	11.93 ± 0.46	28.16 ± 1.96	38.29 ± 1.67	41.78 ± 1.82	53.14 ± 0.98
L990	Iron dextran	6.35 ± 0.27	8.48 ± 0.19*	9.5 ± 0.65*	13.77 ± 0.29*	31.21 ± 1.96*	43.79 ± 1.11*	43.80 ± 2.23	53.00 ± 0.78
	Split	5.90 ± 0.37	8.31 ± 0.18*	8.60 ± 0.56**	13.21 ± 0.19*	28.90 ± 1.66	43.56 ± 0.60*	42.10 ± 1.73	52.43 ± 0.71

Values are expressed as the means ± SE. RBC indices were determined for seven pigs of each treatment group. *RBC* red blood cell count, *HGB* hemoglobin level, *HCT* hematocrit, *MCV* mean corpuscular volume, *RDW* red blood cell distribution width, *MCH* mean corpuscular hemoglobin. *P < 0.05, significant differences between PLW and L990 pigs within a given supplementation group. **P < 0.05, significant differences between pigs receiving split and routine supplementation within a given breed

**Fig. 4:**
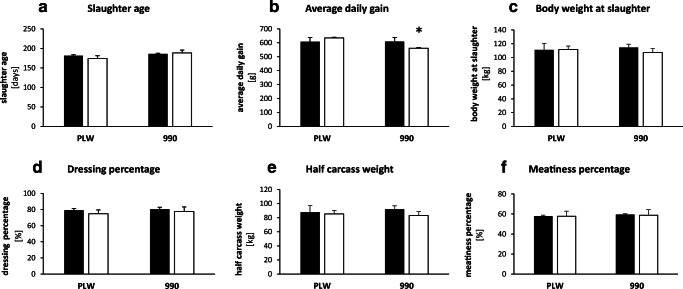
Growth performance and carcass parameters in PLW and L990 ~ 180-day-old pigs receiving various iron supplementations: (**a**) slaughter age; (**b**) average daily gain; (**c**) body weight at slaughter; (**d**) dressing percentage; (**e**) half carcass weight; and (**f**) meatiness percentage. Values are expressed as the means ± SE for pigs of each group (*N* = 7). *P < 0.05, significant differences between pigs receiving split and routine supplementation within a given breed. Solid bars, piglets supplemented with high-dose FeDex; open bars, piglets receiving split FeDex/hemoglobin supplementation

**Fig. 5:**
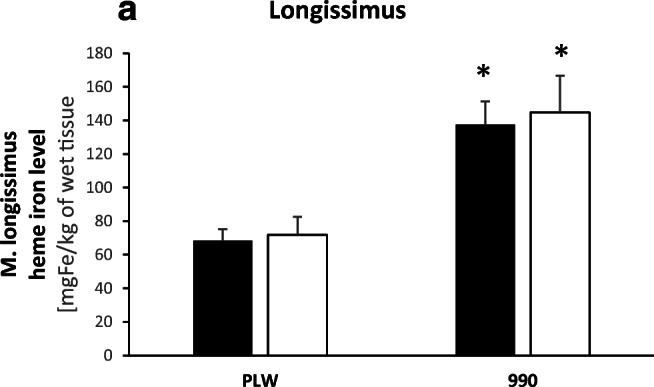
Comparison of the *longissimus thoracis et lumborum* muscle heme iron content in PLW and L990 ~ 180-day-old pigs receiving various iron supplementations. Musculoskeletal heme iron content was measured in (**a**) *longissimus thoracis et lumborum* muscle. Values are expressed as the means ± SE for pigs of each group (*N* = 7). *P < 0.05, significant differences between PLW and L990 pigs within a given supplementation group. Solid bars, piglets supplemented with high-dose FeDex; open bars, piglets receiving split FeDex/hemoglobin supplementation

Considering that pork meat is a valuable source of iron for consumers, it is noteworthy that split supplementation did not result in lower iron content of skeletal muscles such as *musculus longissimus thoracis et lumborum* and *musculus gluteus maximus* in PLW and L990 pigs compared with commonly used high FeDex supplementation (Figs. 3d, e and 5). The slaughter age of pigs was similar among treatments and breeds (Fig. 4a). Comparing to classical supplementation with high dose of FeDex, split FeDex/hemoglobin procedure supported similar average daily gain (Fig. 4b) and final body weight of PLW pigs (Fig. 4c). However, in L990 pigs receiving split supplementation, average daily gain was significantly lower (*P* < 0.05) suggesting that this supplementation does not fully support the fast growth of these animals [40–42]. Apparently, basal iron content in diets for grower-finisher pigs is largely sufficient to compensate for moderate deficiency at weaning and to sustain both normal growth and slaughter performance in PLW but not in L990. It is highly probable that this phenomenon is associated with the high iron demand of this breed for erythropoiesis. Red blood cell indices in L990 piglets/pigs are significantly higher compared with PLW animals (Table 1). Apart from high erythroid iron acquisition, greater need for iron in L990 piglets may result from the use of large amounts of this microelement to sustain heme and myoglobin synthesis in muscle tissue (see Fig. 5 heme iron content). Iron transport to the bone marrow is dependent on endocytosis of diferric transferrin (Tf-Fe_2_) via the transferrin receptor 1 (TFR1) abundantly expressed on erythroid cell [39]. The preferential movement of iron to the bone marrow (at the expense of other tissues) may result in a lower content of this biometal in the liver and kidney (Fig. 3) and may be also responsible for a slight growth retardation (Fig. 4).

Importantly, addition of iron to post-weaning basal diets increasing its content above NRC requirements [43, 44] has been shown dispensable for the improvement of growth performance of pigs, carcass characteristics, and pork quality [21, 45]. However, in contrast to elemental iron (ferrous sulfate), supplemental heme iron given to pigs starting from week 5 after birth favored greater body weight of animals on week 20 after birth suggesting better bioavailability of heme iron than that of elemental iron [46]. It is well known that meat color is a relevant factor that affects meat quality [47]. Meat color is mainly determined by myoglobin, which is the dominant pigment in the skeletal muscle [48]. Interestingly, supplemental heme has been also shown to increase the redness and myoglobin level (which is the main source of heme iron in skeletal muscles) of *longissimus thoracis et lumborum* muscle of 2-month-old piglets when compared with ferrous sulfate as elemental iron source [46].

According to Blicharski et al. (2014) [47] and Borzuta and Lisiak (2016) [49] and as shown in this study (Fig. 4), carcass parameters of PLW and L990 pigs traditionally supplemented with FeDex do not differ significantly. Here, in addition, we show that main meat quality parameters such as loin “eye” area (LEA), color/brightness of meat, backfat thickness, and heme iron level of *longissimus thoracis et lumborum* in pigs of both breeds receiving split FeDex/heme supplementation are very similar to those measured in at slaughter in animals traditionally supplemented with high amount of FeDex (Table 2 and Fig. 5). Therefore, it is tempting to propose that split supplementation could be recommended for use on a larger scale in pig production.

**Table 2:** Backfat thickness from 5 measurements and selected meat quality parameters in PLW and L990 ~ 180-day-old pigs receiving various iron supplementations

Breed ↓	Parameter → Supplementation ↓	FT1 (cm)	FT2 (cm)	FT3 (cm)	FT4 (cm)	FT5 (cm)	FTav (cm)	LEA (cm^2^)	Color brightness (%)
PLW	Iron dextran	3.09 ± 0.42	1.88 ± 0.19	2.36 ± 0.38	1.38 ± 0.12	1.97 ± 0.03	2.16	57.60 ± 2.27	53.48 ± 1.21
	Split	2.87 ± 0.38	1.70 ± 1.03	2.16 ± 0.12	1.33 ± 0.11	2.03 ± 0.07	2.02	53.14 ± 2.19	54.68 ± 1.42
L990	Iron dextran	3.29 ± 0.19	2.04 ± 0.53	2.49 ± 0.12	1.47 ± 0.09	2.23 ± 0.16	2.32	56.79 ± 2.07	55.40 ± 1.17
	Split	3.21 ± 0.20	1.93 ± 0.56	2.45 ± 0.19	1.30 ± 0.08	1.83 ± 0.12	2.14	55.73 ± 1.96	55.68 ± 2.18

Animal quality traits were measured as described in the “Materials and Methods” section. Values are expressed as the means ± SE for pigs of each group (*N* = 7)

## Conclusion

Summing up, our data clearly demonstrate that the combine supplementation of piglets during pre-weaning period with a small amount of intramuscularly injected FeDex and orally given hemoglobin (up to day 45 after birth) is a procedure that warrants further normal growth performance of animals up to slaughter as well as assures high quality of pork. Even though this split supplementation does not fully rebalance RBC and iron status of piglets at weaning, it paves the way for normal pig development under standard iron feeding conditions (from birth until the weaning and at the end of fattening). We postulate that exclusive oral supplementation of suckling piglets with highly available heme iron supplements (even without small amount of intramuscularly injected FeDex) is worth considering for pig breeders. Despite its poorer efficiency in rectifying iron status at weaning compared with classical supplementation with FeDex. We believe that elimination of the supplementation of piglets with FeDex injected intramuscularly will allow to avoid several inconveniences of this routine procedure such as invasiveness, pain, and high labor consumption.
